# Neural stages of spoken, written, and signed word processing in beginning second language learners

**DOI:** 10.3389/fnhum.2013.00322

**Published:** 2013-07-02

**Authors:** Matthew K. Leonard, Naja Ferjan Ramirez, Christina Torres, Marla Hatrak, Rachel I. Mayberry, Eric Halgren

**Affiliations:** ^1^Department of Radiology, University of CaliforniaSan Diego, La Jolla, CA, USA; ^2^Multimodal Imaging Laboratory, Department of Radiology, University of CaliforniaSan Diego, La Jolla, CA, USA; ^3^Department of Linguistics, University of CaliforniaSan Diego, La Jolla, CA, USA; ^4^Department of Neurosciences, University of CaliforniaSan Diego, La Jolla, CA, USA; ^5^Kavli Institute for Brain and Mind, University of CaliforniaSan Diego, La Jolla, CA, USA

**Keywords:** second language acquisition, sign language, speech, reading, proficiency, modality, magnetoencephalography, MRI

## Abstract

We combined magnetoencephalography (MEG) and magnetic resonance imaging (MRI) to examine how sensory modality, language type, and language proficiency interact during two fundamental stages of word processing: (1) an early word encoding stage, and (2) a later supramodal lexico-semantic stage. Adult native English speakers who were learning American Sign Language (ASL) performed a semantic task for spoken and written English words, and ASL signs. During the early time window, written words evoked responses in left ventral occipitotemporal cortex, and spoken words in left superior temporal cortex. Signed words evoked activity in right intraparietal sulcus that was marginally greater than for written words. During the later time window, all three types of words showed significant activity in the classical left fronto-temporal language network, the first demonstration of such activity in individuals with so little second language (L2) instruction in sign. In addition, a dissociation between semantic congruity effects and overall MEG response magnitude for ASL responses suggested shallower and more effortful processing, presumably reflecting novice L2 learning. Consistent with previous research on non-dominant language processing in spoken languages, the L2 ASL learners also showed recruitment of right hemisphere and lateral occipital cortex. These results demonstrate that late lexico-semantic processing utilizes a common substrate, independent of modality, and that proficiency effects in sign language are comparable to those in spoken language.

## Introduction

Humans acquire language in an astonishingly diverse set of circumstances. Nearly everyone learns a spoken language from birth and a majority of individuals then follow this process by learning to read, an extension of their spoken language experience. In contrast to these two tightly-coupled modalities (written words are a visual representation of phonological forms, specific to a given language), there exists another language form that bears no inherent relationship to a spoken form: Sign language. When deaf children are raised by deaf parents and acquire sign as their native language from birth, they develop proficiency within the same time frame and in a similar manner to that of spoken language in hearing individuals (Anderson and Reilly, 2002; Mayberry and Squires, 2006). This is not surprising given that sign languages have sublexical and syntactic complexity similar to spoken languages (Emmorey, 2002; Sandler and Lillo-Martin, 2006). Neural investigations of sign languages have also shown a close correspondence between the processing of signed words in deaf (Petitto et al., 2000; MacSweeney et al., 2008; Mayberry et al., 2011; Leonard et al., 2012) and hearing native signers (MacSweeney et al., 2002, 2006) and spoken words in hearing individuals (many native signers are also fluent in a written language, although the neural basis of reading in deaf individuals is largely unknown). The predominant finding is that left anteroventral temporal, inferior prefrontal, and superior temporal cortex are the main loci of lexico-semantic processing in spoken/written (Marinkovic et al., 2003) and signed languages, as long as the language is learned early or to a high level of proficiency (Mayberry et al., 2011). However, it is unknown whether the same brain areas are used for sign language processing in hearing second language (L2) learners who are beginning to learn sign language. This is a key question for understanding the generalizability of L2 proficiency effects, and more broadly for understanding language mechanisms in the brain.

In contrast to the processing of word meaning, which occurs between ~200–400 ms after the word is seen or heard (Kutas and Federmeier, 2011), processing of the word form and sublexical structure appears to be modality-specific. Written words are encoded for their visual form primarily in left ventral occipitotemporal areas (McCandliss et al., 2003; Vinckier et al., 2007; Dehaene and Cohen, 2011; Price and Devlin, 2011). Spoken words are likewise encoded for their acoustic-phonetic and phonemic forms in left-lateralized superior temporal cortex, including the superior temporal gyrus/sulcus and planum temporale (Hickok and Poeppel, 2007; Price, 2010; DeWitt and Rauschecker, 2012; Travis et al., in press). Both of these processes occur within the first ~170 ms after the word is presented. While an analogous form encoding stage presumably exists with similar timing for sign language, no such process has been identified. The findings from monolingual users of spoken/written and signed languages to date suggest at least two primary stages of word processing: An early, modality-specific word form encoding stage (observed for spoken/written words and hypothesized for sign), followed by a longer latency response that converges on the classical left fronto-temporal language network where meaning is extracted and integrated independent of the original spoken, written, or signed form (Leonard et al., 2012).

Much of the world's population is at least passingly familiar with more than one language, which provides a separate set of circumstances for learning and using words. Often, an L2 is acquired later with ultimately lower proficiency compared to the native language. Fluent, balanced speakers of two or more languages have little difficulty producing words in the contextually correct language, and they understand words as rapidly and efficiently as words in their native language (Duñabeitia et al., 2010). However, prior to fluent understanding, the brain appears to go through a learning process that uses the native language as a scaffold, but diverges in subtle, yet important ways from native language processing. The extent of these differences (both behaviorally and neurally) fluctuates in relation to the age at which L2 learning begins, the proficiency level at any given moment during L2 learning, the amount of time spent using each language throughout the course of the day, and possibly the modality of the newly-learned language (DeKeyser and Larson-Hall, 2005; van Heuven and Dijkstra, 2010). Thus, L2 learning provides a unique opportunity to examine the role of experience in how the brain processes words.

In agreement with many L2 speakers' intuitive experiences, several behavioral studies using cross-language translation priming have found that proficiency and language dominance impact the extent and direction of priming (Basnight-Brown and Altarriba, 2007; Duñabeitia et al., 2010; Dimitropoulou et al., 2011). The most common finding is that priming is strongest in the dominant to non-dominant direction, although the opposite pattern has been observed (Duyck and Warlop, 2009). These results are consistent with models of bilingual lexical representations, including the Revised Hierarchical Model (Kroll and Stewart, 1994) and the Bilingual Interactive Activation + (BIA+) model (Dijkstra and van Heuven, 2002), both of which posit interactive and asymmetric connections between word (and sublexical) representations in both languages. The BIA+ model is particularly relevant here, in that it explains the proficiency-related differences as levels of activation of the integrated (i.e., shared) lexicon driven by the bottom-up input of phonological/orthographic and word-form representations.

An important question is how these behavioral proficiency effects manifest in neural activity patterns: Does the brain process less proficient words differently from more familiar words? Extensive neuroimaging and neurophysiological evidence supports these models, and shows a particularly strong role for proficiency in cortical organization (van Heuven and Dijkstra, 2010). Two recent studies that measured neural activity with magnetoencephalography (MEG) constrained by individual subject anatomy obtained with magnetic resonance imaging (MRI) found that, while both languages for Spanish-English bilinguals evoked activity in the classical left hemisphere fronto-temporal network, the non-dominant language additionally recruited posterior and right hemisphere regions (Leonard et al., 2010, 2011). These areas showed significant non-dominant > dominant activity during an early stage of word encoding (between ~100–200 ms), continuing through the time period typically associated with lexico-semantic processing (~200–400 ms). Crucially, these and other studies (e.g., van Heuven and Dijkstra, 2010) showed that language proficiency was the main factor in determining the recruitment of non-classical language areas. The order in which the languages were acquired did not greatly affect the activity.

These findings are consistent with the hemodynamic imaging and electrophysiological literatures. Using functional MRI (fMRI), proficiency-modulated differences in activity have been observed (Abutalebi et al., 2001; Chee et al., 2001; Perani and Abutalebi, 2005), and there is evidence for greater right hemisphere activity when processing the less proficient L2 (Dehaene et al., 1997; Meschyan and Hernandez, 2006). While fMRI provides spatial resolution on the order of millimeters, the hemodynamic response unfolds over the course of several seconds, far slower than the time course of linguistic processing in the brain. Electroencephalographic methods including event-related potentials (ERPs) are useful for elucidating the timing of activity, and numerous studies have found proficiency-related differences between bilinguals' two languages. One measure of lexico-semantic processing, the N400 [or N400 m in MEG; (Kutas and Federmeier, 2011)] is delayed by ~40–50 ms in the L2 (Ardal et al., 1990; Weber-Fox and Neville, 1996; Hahne, 2001), and this effect is constrained by language dominance (Moreno and Kutas, 2005), in agreement with the behavioral and MEG studies discussed above. In general, greater occipito-temporal activity in the non-dominant language (particularly on the right), viewed in light of delayed processing, suggests that lower proficiency involves less efficient processing that requires recruitment of greater neural resources. While the exact neural coding mechanism is not known, this is a well-established phenomenon that applies to both non-linguistic (Carpenter et al., 1999) and high-level language tasks (St George et al., 1999) at the neuronal population level.

The research to date thus demonstrates two main findings: (1) In nearly all subject populations that have been examined, lexico-semantic processing is largely unaffected by language modality with respect to spoken, written, and signed language, and (2) lower proficiency involves the recruitment of a network of non-classical language regions that likewise appear to be modality-independent. In the present study, we sought to determine whether the effects of language proficiency extend to hearing individuals who are learning sign language as an L2. Although these individuals have extensive experience with a visual language form (written words), their highly limited exposure to dynamic sign language forms allows us to investigate proficiency (English vs. ASL) and modality (spoken vs. written, vs. signed) effects in a single subject population. We tested a group of individuals with a unique set of circumstances as they relate to these two factors. The subjects were undergraduate students who were native English speakers who began learning American Sign Language (ASL) as an L2 in college. They had at least 40 weeks of experience, and were the top academic performers in their ASL courses and hence able to understand simple ASL signs and phrases. They were, however, unbalanced bilinguals with respect to English/ASL proficiency. Although there have been a few previous investigations of highly proficient, hearing L2 signers (Neville et al., 1997; Newman et al., 2001), no studies have investigated sign language processing in L2 learners with so little instruction. Likewise, no studies have investigated this question using methods that afford high spatiotemporal resolution to determine both the cortical sources and timing of activity during specific processing stages. Similar to our previous studies on hearing bilinguals with two spoken languages, here we combined MEG and structural MRI to examine neural activity in these subjects while they performed a semantic task in two languages/modalities: spoken English, visual (written) English, and visual ASL.

While it is not possible to fully disentangle modality and proficiency effects within a single subject population, these factors have been systematically varied separately in numerous studies with cross-language and between-group comparisons (Marinkovic et al., 2003; Leonard et al., 2010, 2011, 2012), and are well-characterized in isolation. It is in this context that we examined both factors in this group of L2 learners. We hypothesized that a comparison between the magnitudes of MEG responses to spoken, written, and signed words would reveal a modality-specific word encoding stage between ~100–200 ms (left superior planar regions for spoken words, left ventral occipitotemporal regions for written words, and an unknown set of regions for signed words), followed by stronger responses for ASL (the lower proficiency language) in a more extended network of brain regions used to process lexico-semantic content between ~200–400 ms post-stimulus onset. These areas have previously been identified in spoken language L2 learners and include bilateral posterior visual and superior temporal areas (Leonard et al., 2010, 2011). Finding similar patterns for beginning ASL L2 learners would provide novel evidence that linguistic proficiency effects are generalizable, a particularly striking result given the vastly different sensory characteristics of spoken English and ASL. We further characterized the nature of lexico-semantic processing in this group by comparing the N400 effect across modalities, which would reveal differences in the loci of contextual integration for relatively inexperienced learners of a visual second language.

## Materials and methods

### Participants

Eleven hearing native English speakers participated in this study (10 F; age range = 19.74–33.16 years, mean = 22.42). All were healthy adults with no history of neurological or psychological impairment, and had normal hearing and vision (or wore corrective lenses that were applied in the MEG). All participants had at least four academic quarters (40 weeks) of instruction in ASL, having reached the highest level of instruction at either UCSD or Mesa College. Participants were either currently enrolled in a course taught in ASL or had been enrolled in such a course in the previous month. One participant had not taken an ASL course in the previous 4 months. Participants completed a self-assessment questionnaire that asked them to rate their ASL proficiency on a scale from 1 to 10. For ASL comprehension, the average score was 7.1 ± 1.2; ASL production was 6.5 ± 1.9; Fingerspelling comprehension was 6.4 ± 1.6; and fingerspelling production was 6.8 ± 1.7. Six participants reported using ASL on a daily basis at the time of enrollment in the study, while the remaining participants indicated weekly use (one participant indicated monthly use).

Participants gave written informed consent to participate in the study, and were paid $20/h for their time. This study was approved by the Institutional Review Board at the University of California, San Diego.

### Stimuli and procedure

In the MEG, participants performed a semantic decision task that involved detecting a match in meaning between a picture and a word. For each trial, subjects saw a photograph of an object for 700 ms, followed by a word that either matched (“congruent”) or mismatched (“incongruent”) the picture in meaning. Participants were instructed to press a button when there was a match; response hand was counterbalanced across blocks within subjects. Words were presented in blocks by language/modality for spoken English, written English, and ASL. Each word appeared once in the congruent and once in the incongruent condition, and did not repeat across modalities. All words were highly imageable concrete nouns that were familiar to the participants in both languages. Since no frequency norms exist for ASL, the stimuli were selected from ASL developmental inventories (Schick, 1997; Anderson and Reilly, 2002) and picture naming data (Bates et al., 2003; Ferjan Ramirez et al., 2013b). The ASL stimuli were piloted with four other subjects who had the same type of ASL instruction to confirm that they were familiar with the words. Stimulus length was the following: Spoken English mean = 473.98 ± 53.17 ms; Written English mean = 4.21 ± 0.86 letters; ASL video clips mean = 467.92 ± 62.88 ms. Written words appeared on the screen for 1500 ms. Auditory stimuli were delivered through earphones at an average amplitude of 65 dB SPL. Written and signed word videos subtended <5 degrees of visual angle on a screen in front of the subjects. For all stimulus types, the total trial duration varied randomly between 2600 and 2800 ms (700 ms picture + 1500 ms word container + 400–600 ms inter-trial interval).

Each participant completed three blocks of stimuli in each language/modality. Each block had 100 trials (50 stimuli in each of the congruent and incongruent conditions) for a total of 150 congruent and incongruent trials in each language/modality. The order of the languages/modalities was counterbalanced across participants. Prior to starting the first block in each language/modality, participants performed a practice run to ensure they understood the stimuli and task. The practice runs were repeated as necessary until subjects were confident in their performance (no subjects required more than one repetition of the practice blocks).

### MEG recording

Participants sat in a magnetically shielded room (IMEDCO-AG, Switzerland) with the head in a Neuromag Vectorview helmet-shaped dewar containing 102 magnetometers and 204 gradiometers (Elekta AB, Helsinki, Finland). Data were collected at a continuous sampling rate of 1000 Hz with minimal filtering (0.1 to 200 Hz). The positions of four non-magnetic coils affixed to the subjects' heads were digitized along with the main fiduciary points such as the nose, nasion, and preauricular points for subsequent coregistration with high-resolution MR images. The average 3-dimensional Euclidian distance for head movement from the beginning of the session to the end of the session was 7.38 mm (SD = 5.67 mm).

### Anatomically-constrained MEG (aMEG) analysis

The data were analyzed using a multimodal imaging approach that constrains the MEG activity to the cortical surface as determined by high-resolution structural MRI (Dale et al., 2000). This noise-normalized linear inverse technique, known as dynamic statistical parametric mapping (dSPM) has been used extensively across a variety of paradigms, particularly language tasks that benefit from a distributed source analysis (Marinkovic et al., 2003; Leonard et al., 2010, 2011, 2012; Travis et al., in press), and has been validated by direct intracranial recordings (Halgren et al., 1994; McDonald et al., 2010).

The cortical surface was obtained in each participant with a T1-weighted structural MRI, and was reconstructed using FreeSurfer. The images were collected at the UCSD Radiology Imaging Laboratory with a 1.5T GE Signa HDx scanner using an eight-channel head coil (*TR* = 9.8 ms, *TE* = 4.1 ms, *TI* = 270 ms, flip angle = 8°, bandwidth = ± 15.63 kHz, FOV = 24 cm, matrix = 192 × 192, voxel size = 1.25 × 1.25 × 1.2 mm). All T1 scans were collected using online prospective motion correction (White et al., 2010). A boundary element method forward solution was derived from the inner skull boundary (Oostendorp and Van Oosterom, 1992), and the cortical surface was downsampled to ~2500 dipole locations per hemisphere (Dale et al., 1999; Fischl et al., 1999). The orientation-unconstrained MEG activity of each dipole was estimated every 4 ms, and the noise sensitivity at each location was estimated from the average pre-stimulus baseline from −190 to −20 ms for the localization of the subtraction for congruent-incongruent trials.

The data were inspected for bad channels (channels with excessive noise, no signal, or unexplained artifacts), which were excluded from all further analyses. Additionally, trials with large (>3000 fT for gradiometers) transients were rejected. Blink artifacts were removed using independent components analysis (Delorme and Makeig, 2004) by pairing each MEG channel with the electrooculogram (EOG) channel, and rejecting the independent component that contained the blink. On average, fewer than five trials were rejected for each condition.

Individual participant dSPMs were constructed from the averaged data in the trial epoch for each condition using only data from the gradiometers, and then these data were combined across subjects by taking the mean activity at each vertex on the cortical surface and plotting it on an average brain. Vertices were matched across subjects by morphing the reconstructed cortical surfaces into a common sphere, optimally matching gyral-sulcal patterns and minimizing shear (Sereno et al., 1996; Fischl et al., 1999).

**Figure 1:**
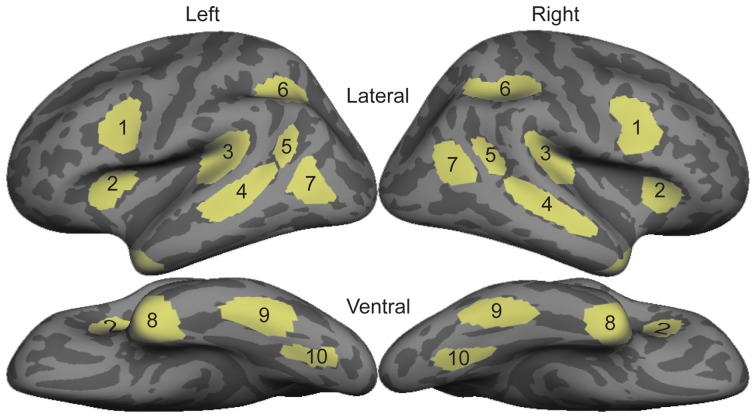
**Diagram of bilateral ROI locations**. These ROIs were selected based on an independent data set that compared sign in native deaf individuals to speech in hearing individuals (Leonard et al., 2012). 1, Inferior Prefrontal; 2, Anterior Insula; 3, Planum Temporale; 4, Superior Temporal Sulcus; 5, Posterior STS; 6, Intraparietal Sulcus; 7, Lateral Occipito-temporal cortex; 8, Temporal Pole; 9, Inferior Temporal; 10, Ventral Occipito-temporal cortex.

All statistical comparisons were made on region of interest (ROI) timecourses from these group data. ROIs were based on a separate data set not included in this study that compared signed and spoken word processing in congenitally deaf and hearing subjects using the same task presented here (Figure 1; Leonard et al., 2012). These ROIs were originally drawn on the grand average activity across both deaf and hearing participants, and thus are not biased toward either signed or spoken words. In the 80–120 ms time window, we specifically tested bilateral planum temporale (PT) and superior temporal sulcus (STS) because these areas showed significant responses to spoken words, and are known to be involved in early word encoding in the auditory modality (Uusvuori et al., 2008; Travis et al., in press). For the 150–200 ms time window, we were specifically interested in ventral occipitotemporal (vOT) cortex because it is involved in written word form encoding (Vinckier et al., 2007). While there are no previous studies of this stage for signed words, we selected bilateral intraparietal sulcus (IPS) because it has been implicated in some studies of non-temporally specific sign processing (MacSweeney et al., 2002; Emmorey et al., 2005), and because it showed the strongest activity during this time window. For the lexico-semantic time window from 300 to 400 ms, we tested all ten bilateral ROIs. With the exceptions of IPS and lateral occipitotemporal (LOT) cortex, these areas are typically involved in lexico-semantic processing, including anteroventral temporal areas that are hypothesized to be largely supramodal. We also included LOT because it has been implicated as a lexico-semantic area that is modulated by proficiency (Leonard et al., 2010, 2011).

## Results

### Reaction time and accuracy

The following analyses use these abbreviations: **A = auditory words, W = written words, and S = signed words**. Participants performed within ranges similar to those of native speakers and signers on the semantic decision task in both reaction time and accuracy compared to results in other studies (Leonard et al., 2012; Travis et al., in press). Table 1 shows the average and standard deviations for each language/modality. A one-way ANOVA comparing reaction times across modalities revealed a significant effect of modality [*F*_(2, 30)_ = 12.21, *p* < 0.0002]. Consistent with the fact that English was the subjects' native and dominant language and ASL was a recently learned L2, reaction times were significantly faster for A than for S [*t*_(10)_ = 6.85, *p* < 0.0001], and for W than for S [*t*_(10)_ = 8.22, *p* < 0.0001]. A and W were not significantly different. Similarly, there was a significant effect of modality for accuracy on the semantic task [*F*_(2, 30)_ = 17.31, *p* < 0.00001]. Participants were more accurate for A than for S [*t*_(10)_ = 5.13, *p* < 0.0001], and for W than for S [*t*_(10)_ = 4.13, *p* = 0.002], although accuracy for S was still quite good (nearly 90%). Accuracy for A and W were not significantly different.

**Table 1:** **Mean reaction time and accuracy data across languages/modalities**.

	**Language/modality**		
	**Spoken English**	**Written English**	**ASL**
RT (SD) ms	582.64 (77.51)	542.80 (92.81)	719.49 (92.67)
Accuracy (SD) %	97.91 (1.14)	96.00 (3.46)	89.36 (5.01)

### aMEG results summary

**Figure 2:**
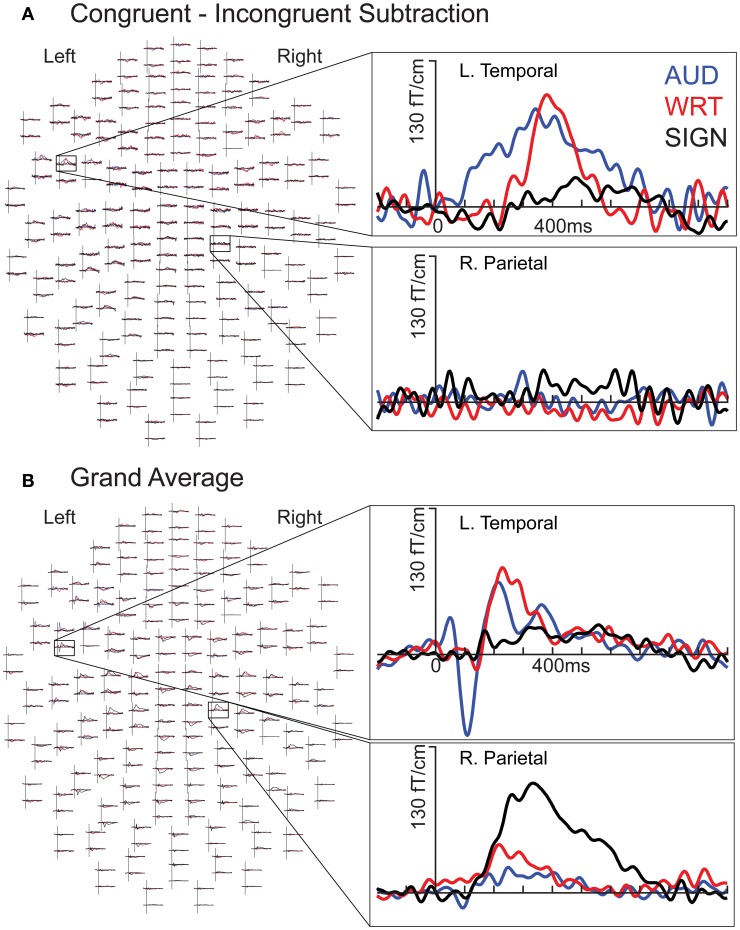
**Individual subject waveforms showing sensor-level language/modality effects. (A)** Congruent-Incongruent waveforms for each modality for a left temporal channel (top) show greater responses for auditory (“AUD”; blue) and written (“WRT”; red) than for signed (“SIGN”; black) words. In a right parietal channel (bottom), there are no strong responses in any condition. **(B)** Grand average waveforms for each modality for a left temporal channel (top) show an early word encoding peak at ~100 ms for auditory words, followed by overlap between all three conditions at ~400 ms. In the same right parietal channel (bottom), signed words evoke an early and persistent response that is stronger than the responses for both English modalities.

There were distinct patterns of neural activity related to the language/modality subjects saw or heard and language proficiency. These effects began during the earliest stages of word encoding (~100 ms for auditory, and ~150 ms for written and signed words), and continued through lexico-semantic encoding (~300–400 ms). Table 2 summarizes the main findings by time window, and the following sections statistically describe the effects shown in the table and figures. Figure 2 shows sensor-level data from a single representative subject.

**Table 2:** **Summary of MEG effects by time window**.

**Effect**	**Time window**		
	**Early encoding**	**Lexico-semantic (congruity)**	**Lexico-semantic (overall)**
A > W & S	L PT	R STS	
	L STS		
	R STS		
W > A & S	L vOT		
S > A & W	R IPS		L IFG
			L IT
			R IFG
			R IPS
			R LOT
			R PT
			R pSTS
A & W > S		L IFG	
		L IT	
		L pSTS	
		L PT	
		L STS	
		R IFG	

### aMEG—80−120 ms (early word encoding)

**Figure 3:**
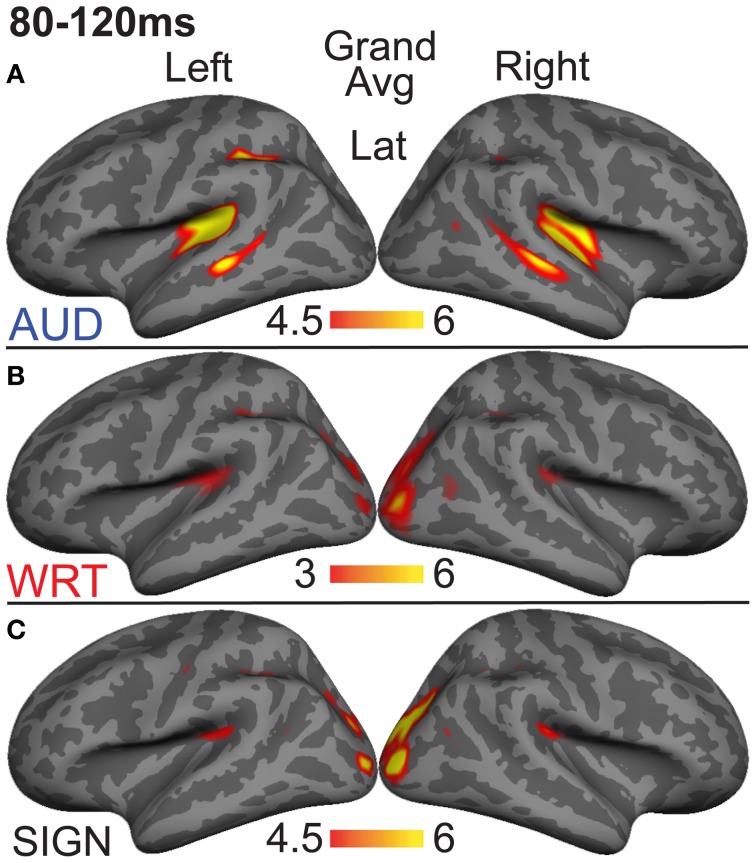
**Grand average group dSPMs during the early encoding time window from 80 to 120 ms. (A)** Auditory words (“AUD”) show strong responses in bilateral PT and STS. **(B)** Written (“WRT”) and **(C)** signed (“SIGN”) words show sensory processing at the occipital pole. *F*-values on the color bars represent signal-to-noise ratios.

**Figure 4:**
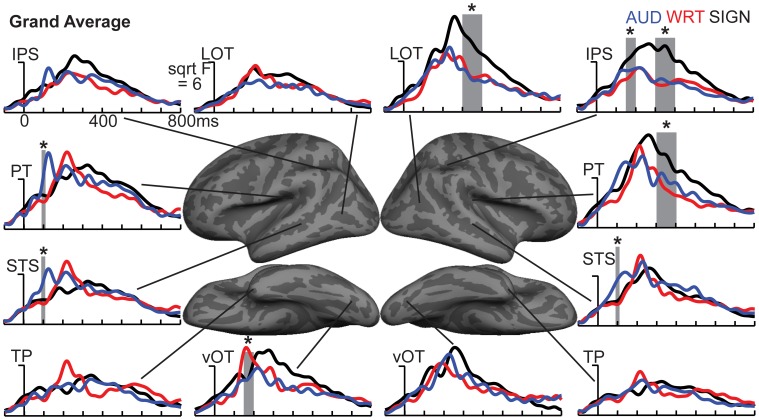
**ROI timecourses for the grand average across each language/modality**. At 80–120 ms in left PT, auditory words (blue lines) show a strong modality-specific peak. From 150 to 200 ms, written words (red lines) show a word encoding peak in left vOT, and signed words (black lines) show a word encoding effect in right IPS. During a later time window from 300 to 400 ms (thick gray bars), all conditions show similar responses in most left hemisphere regions, but signed words show much stronger responses in right hemisphere regions, including LOT, IPS, and PT. Asterisks represent statistically significant differences. Abbreviations: IPS, intraparietal sulcus; LOT, lateral occipitotemporal; PT, planum temporale; STS, superior temporal sulcus; TP, temporal pole; vOT, ventral occipitotemporal.

Previous investigations have identified an evoked potential peak at ~100 ms that shows selectivity for auditory speech stimuli compared to sensory controls in primarily left superior temporal and superior planar cortex (Uusvuori et al., 2008; Travis et al., in press). We tested the MEG response during this window in two areas to determine whether an auditory-selective modality effect was present. We found a main effect of modality in left planum temporale (PT) [*F*_(1, 10)_ = 3.58, *p* = 0.047], and in left superior temporal sulcus (STS) [*F*_(1, 10)_ = 6.22, *p* = 0.008] (Figure 3). The effect in PT was driven by trending A>W [*t*_(10)_ = 1.95, *p* = 0.079] and A>S [*t*_(10)_ = 1.93, *p* = 0.083] responses, and likewise for STS [*t*_(10)_ = 2.77, *p* = 0.02; *t*_(10)_ = 2.37, *p* = 0.039] (Figure 4). Similar effects were obtained in right PT [*F*_(1, 10)_ = 6.15, *p* = 0.008] and STS [*F*_(1, 10)_ = 10.74, *p* = 0.001]. While the right STS effect was driven by an A>W [*t*_(10)_ = 4.00, *p* = 0.003] and A>S [*t*_(10)_ = 2.81, *p* = 0.018] response, the right PT effect showed an overall smaller response to W compared with A [*t*_(10)_ = 3.32, *p* = 0.008] and S [*t*_(10)_ = 3.00, *p* = 0.013]. Thus, during the 80–120 ms time window, the brain showed a preferential response for auditory words primarily in superior temporal areas.

### aMEG—150−200 ms (early word encoding)

**Figure 5:**
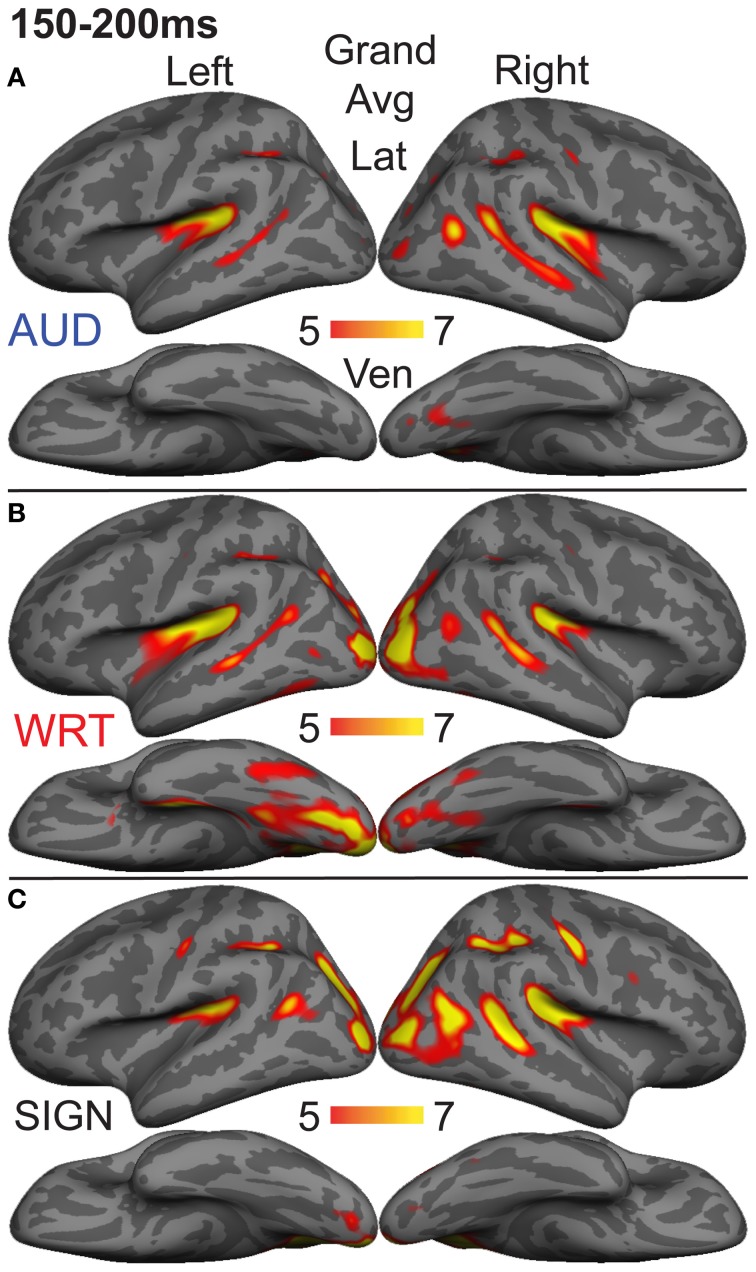
**Grand average group dSPMs during the early encoding time window from 150 to 200 ms. (A)** Auditory words (“AUD”) continue to evoke activity in bilateral superior temporal cortex, while **(B)** Written words (“WRT”) show a modality-specific peak in left vOT. **(C)** Signed words (“SIGN”) show a modality-specific peak in right IPS. *F*-values on the color bars represent signal-to-noise ratios.

The early word encoding response to written words occurs later than for auditory words, and is centered in a left posterior ventral occipitotemporal (vOT) region. During a window from 150 to 200 ms, we tested for a W>A and W>S effect in vOT (Figure 5). In the left hemisphere, there was a main effect of modality [*F*_(1, 10)_ = 4.57, *p* = 0.023], driven by W>A [*t*_(10)_ = 4.58, *p* = 0.001] and W>S [*t*_(10)_ = 2.36, *p* = 0.04] responses (Figure 4). The homologous right hemisphere vOT region did not show significant effects (*p*s > 0.5).

Given that there are early word encoding processes for auditory and written words, it is reasonable to ask whether such a process exists for signed words. We examined the response to signs from 150 to 200 ms, when we expect post-sensory, but pre-lexical processing to occur. The dSPM timecourses in Figure 4 revealed a S>A and S>W pattern in right intraparietal sulcus (IPS), and indeed this region showed a marginal main effect of modality [*F*_(1, 10)_ = 3.20, *p* = 0.062]. *Post-hoc* tests revealed a significant S>W response [*t*_(10)_ = 2.51, *p* = 0.031], but the differences between W & A and S & A were not significant (Figure 5).

### aMEG—300−400 ms (lexico-semantic processing)

Based on results from a previous study that compared sign processing in deaf native signers to spoken word processing in hearing native English speakers using the same task presented here (Leonard et al., 2012), and on previous work examining both early and late processing of spoken vs. written words (Marinkovic et al., 2003), we selected ten ROIs to investigate sensitivity to semantic congruity: Inferior prefrontal cortex, anterior insula, planum temporale, superior temporal sulcus, posterior superior temporal sulcus, intraparietal sulcus, lateral occipitotemporal cortex, temporal pole, inferior temporal cortex, and ventral occipitotemporal cortex (Leonard et al., 2012). For each language and modality, A, W, and S, we calculated dSPMs of the subtraction of incongruent-congruent words, extracted timecourses for each subtraction condition, and tested for within-subject effects of language and modality. Since this procedure isolates brain activity evoked by incongruent vs. congruent trials, it follows that any significant activity indicates the localization of N400-like semantic congruity effects.

We calculated an omnibus ANOVA with three within-subject factors: Language/modality (3), ROI (10), and hemisphere (2). There were highly significant main effects of language/modality [*F*_(2, 20)_ = 6.96, *p* = 0.005], ROI [*F*_(9, 90)_ = 6.76, *p* < 0.0001], and hemisphere [*F*_(1, 10)_ = 10.07, *p* = 0.01]. There were significant interactions between language/modality and ROI [*F*_(18, 180)_ = 2.35, *p* = 0.002], and language/modality and hemisphere [*F*_(2, 20)_ = 9.75, *p* = 0.001], but no three-way interaction.

**Figure 6:**
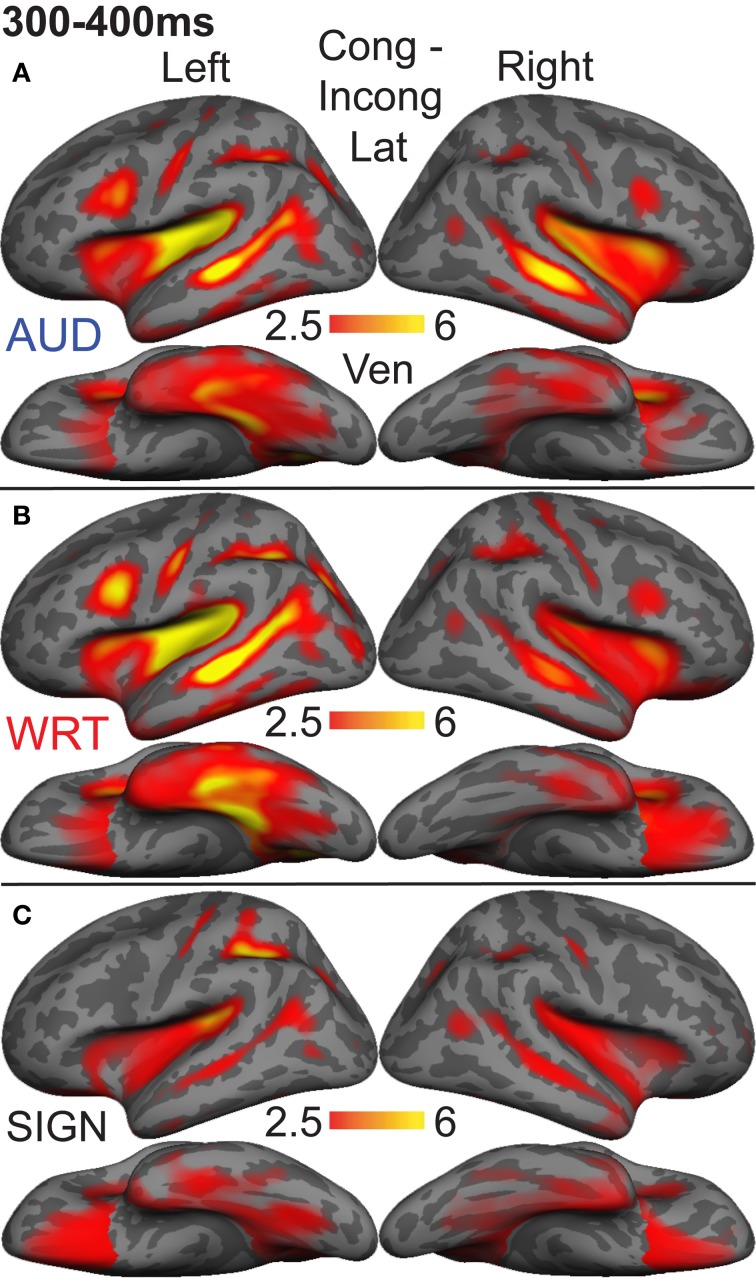
**Congruent-Incongruent subtraction dSPMs during the late lexico-semantic time window from 300 to 400 ms. (A–C)** All three conditions show similar patterns of activity in predominantly left fronto-temporal regions, including PT, STS, inferior frontal, and anteroventral temporal. **(C)** Signed words (“SIGN”) show overall smaller subtraction effects. *F*-values on the color bar represent signal-to-noise ratios.

**Figure 7:**
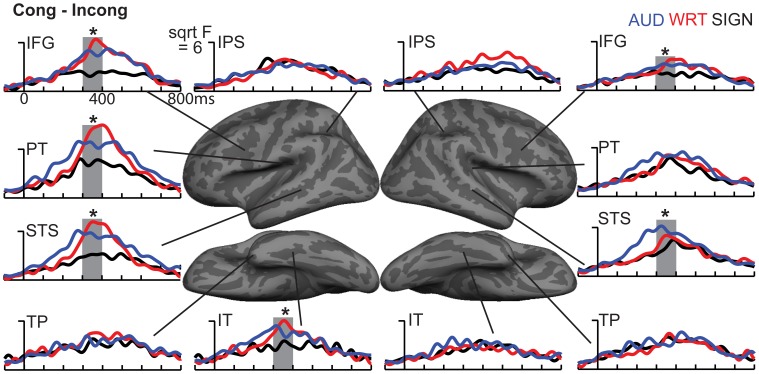
**ROI timecourses for the Congruent-Incongruent subtraction across each language/modality**. From 300 to 400 ms (thick gray bars), auditory (blue lines) and written (red lines) words evoke stronger effects than signed words (black lines). This difference is most prominent in the classical left fronto-temporal language network. Asterisks represent statistically significant differences.

Based on *a priori* hypotheses about specific ROIs from previous studies (see Materials and Methods), we tested a series of planned comparisons across modalities. Overall, there was a highly similar response to A, W, and S words (Figure 6). While A and W showed semantic effects of a similar magnitude, these were weaker for S across most regions (Figure 7). In the left hemisphere, there was a main effect in inferior frontal cortex [*F*_(1, 10)_ = 9.92, *p* = 0.001], driven by A>S [*t*_(10)_ = 3.81, *p* = 0.003] and W>S [*t*_(10)_ = 3.29, *p* = 0.008] responses. Similarly, in inferior temporal (IT) cortex, there was an effect of modality [*F*_(1, 10)_ = 5.94, *p* = 0.009] with A>S [*t*_(10)_ = 2.40, *p* = 0.038] and W>S [*t*_(10)_ = 3.50, *p* = 0.006]. In posterior STS (pSTS), there was a significant difference [*F*_(1, 10)_ = 4.97, *p* = 0.018], driven primarily by a W>S response [*t*_(10)_ = 3.09, *p* = 0.011] and a trend for A>S [*t*_(10)_ = 1.98, *p* = 0.075]. Superior temporal regions showed main effects of modality where all three conditions differed significantly from one another [PT: *F*_(1, 10)_ = 15.03, *p* < 0.0001; STS: *F*_(1, 10)_ = 24.71, *p* < 0.0001]. None of the other five left hemisphere ROIs showed significant differences between language/modality effects.

In the right hemisphere, S elicited smaller responses in inferior frontal cortex [*F*_(1, 10)_ = 4.70, *p* = 0.021], with W>S [*t*_(10)_ = 2.66, *p* = 0.024] and a marginal A>S difference [*t*_(10)_ = 2.14, *p* = 0.058]. In STS, there was a main effect of modality [*F*_(1, 10)_ = 5.68, *p* = 0.011], driven primarily by a strong A>S response [*t*_(10)_ = 3.51, *p* = 0.006] and a trend for A>W [*t*_(10)_ = 1.88, *p* = 0.09]. None of the other eight right hemisphere ROIs showed significant language/modality effects. Thus, lexico-semantic congruity effects occurred in similar areas across languages/modalities, but with a smaller magnitude for signed words.

### aMEG—300−400 ms (overall responses)

**Figure 8:**
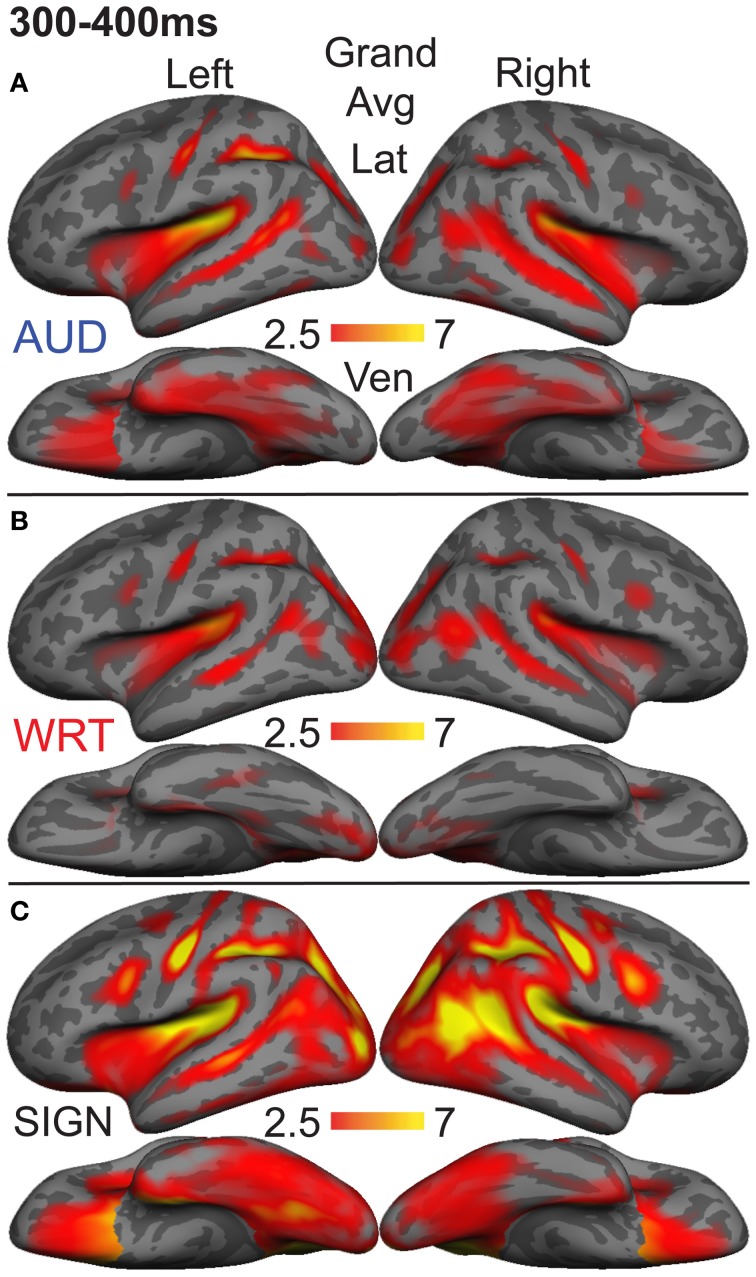
**Grand average group dSPMs during the late lexico-semantic time window from 300 to 400 ms. (A–C)** All three conditions show a similar pattern of activity in bilateral regions. **(C)** Signed words (“SIGN”) show much stronger activity, particularly in the right hemisphere. *F*-values on the color bars represent signal-to-noise ratios.

To understand which regions responded to words in each language/modality, but which were not necessarily influenced by semantic context, we also examined the grand average responses of congruent and incongruent trials together at 300–400 ms. While the previous analysis demonstrated small congruity effects for signed words, examination of the grand average revealed a different pattern (Figure 8). In the same ROIs, we tested these grand averages for language/modality effects (Figure 4).

In the left hemisphere, inferior frontal cortex showed a main effect of language/modality [*F*_(1, 10)_ = 3.65, *p* = 0.044] with S>W [*t*_(10)_ = 2.36, *p* = 0.04] and S>A [*t*_(10)_ = 2.76, *p* = 0.02]. IT showed a similar marginal effect [*F*_(1, 10)_ = 3.35, *p* = 0.056], driven by a marginal S>W effect [*t*_(10)_ = 2.21, *p* = 0.052] and a trend for S>A [*t*_(10)_ = 2.05, *p* = 0.067]. None of the other eight left hemisphere ROIs showed significant language/modality effects.

In the right hemisphere, we observed widespread effects where signs evoked greater activity than auditory or written words. Inferior frontal cortex showed this pattern [*F*_(1, 10)_ = 10.78, *p* = 0.001] with S>W [*t*_(10)_ = 3.19, *p* = 0.01] and S>A [*t*_(10)_ = 3.85, *p* = 0.003]. The same pattern was found for IPS [*F*_(1, 10)_ = 19.81, *p* < 0.0001] with S>W [*t*_(10)_ = 7.03, *p* < 0.0001] and S>A [*t*_(10)_ = 3.85, *p* = 0.003]. In lateral occipitotemporal (LOT) cortex, there was a main effect of language/modality [*F*_(1, 10)_ = 6.21, *p* = 0.008] with S>W [*t*_(10)_ = 2.89, *p* = 0.016] and S>A [*t*_(10)_ = 2.62, *p* = 0.026]. Similarly, language/modality effects were apparent in PT ([*F*_(1, 10)_ = 5.09, *p* = 0.016] with S>W [*t*_(10)_ = 2.76, *p* = 0.02] and S>A [*t*_(10)_ = 2.44, *p* = 0.035]) and in pSTS ([*F*_(1, 10)_ = 4.97, *p* = 0.018] with S>W [*t*_(10)_ = 3.38, *p* = 0.007] and S>A [*t*_(10)_ = 2.01, *p* = 0.072]). The other five right hemisphere ROIs did not show significant language/modality effects. To summarize, although all languages/modalities showed similar lexico-semantic congruity effects in the classical left fronto-temporal language network, the overall response magnitude to signed words was greater primarily in right hemisphere regions.

**Figure 9:**
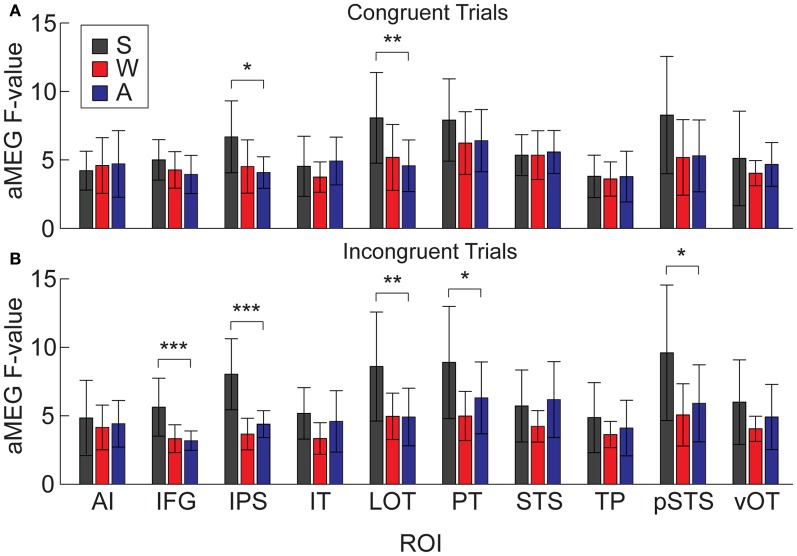
**Mean dSPM values for all 10 right hemisphere ROIs, analyzed separately for congruent and incongruent trials. (A)** One-Way ANOVAs testing for differences across modalities on congruent trials were significant in IPS and LOT. **(B)** Effects for incongruent trials were significant in IFG, IPS, LOT, PT, and pSTS. ^*^p < 0.05; ^**^p < 0.01; ^***^p < 0.001.

It is possible that the overall aMEG responses contain a bias when looking at between-modality differences if the effects are not of similar magnitudes for both congruent and incongruent trials. Therefore, we conducted an additional analysis that compared S, W, and A words for congruent and incongruent trials separately (Figure 9). The one-way ANOVAs for each ROI showed that there were significant differences for congruent trials in right IPS [*F*_(2, 30)_ = 5.33, *p* = 0.01] and right LOT [*F*_(2, 30)_ = 5.68, *p* = 0.008]. For incongruent trials, the same pattern was significant for the following right hemisphere ROIs: IPS [*F*_(2, 30)_ = 20.07, *p* < 0.0001], LOT [*F*_(2, 30)_ = 6.36, *p* = 0.005], IFG [*F*_(2, 30)_ = 10.37, *p* = 0.0004], PT [*F*_(2, 30)_ = 4.84, *p* = 0.015], and pSTS [*F*_(2, 30)_ = 5.116, *p* = 0.01]. Thus, the right hemisphere effects we observed with the combined congruent/incongruent grand average are observed consistently in analyses with only incongruent trials, and also for only congruent trials in two ROIs.

## Discussion

In the present study we examined the spatiotemporal dynamics of word processing across spoken and written English and ASL in a group of hearing, English native speakers who were beginning L2 learners of ASL. During an early word encoding stage (~100 ms for spoken English, and ~150 ms for written English and ASL), words evoked activity in modality-specific brain regions. Responses to English words in the auditory and visual modalities conformed to previous findings in superior temporal and ventral occipitotemporal areas, respectively. ASL signs evoked a strong response in right IPS, although the activity was only marginally significantly larger than for written and spoken English. During a later time window associated with lexico-semantic processing, a distributed network of bilateral regions responded to a semantic congruity manipulation. Several classical left fronto-temporal language areas showed stronger modulation for English (the native language) in spoken and written modalities relative to the L2, ASL. However, when we examined the overall activity during this time window, by disregarding congruity effects, signed words evoked greater activity than both spoken and written words in a network of mostly right hemisphere regions. See Table 2 for a summary of the results.

The early modality-specific word encoding responses are consistent with a large number of previous studies using a variety of methodologies. For written words, we observed a peak in left vOT, a region that has been shown to be important for reading, and specifically for constructing written word-forms (McCandliss et al., 2003; Vinckier et al., 2007; Dehaene and Cohen, 2011; Price and Devlin, 2011). Although there is evidence that it is a multi-modal region (Price and Devlin, 2003), it does seem to play an important role in encoding written words. In addition to the location, the peak timing of the activity in this region at ~170 ms is consistent with previous electrophysiological and neuroimaging studies (McCandliss et al., 2003; McDonald et al., 2010). Additionally, although written and signed words are perceived through the visual modality, signs did not evoke activity in this region in this group of beginning L2 learners of ASL. It is therefore possible that early encoding activity in left vOT is specific to static written word forms.

Also consistent with previous studies, we observed that areas typically associated with encoding spoken words include a bilateral network of superior temporal and superior planar regions (Hickok and Poeppel, 2007; Price, 2010). Many of these areas are sensitive to subtle sublexical and phonetic manipulations (Uusvuori et al., 2008) including the presence of the fundamental frequency (Parviainen et al., 2005) and alterations in voice-onset time (Frye et al., 2007). Specific neural populations within superior temporal cortex have been found to encode categorical and phoneme-selective information within the first ~150 ms (Chang et al., 2010; Travis et al., in press). While the mechanisms and specific representations in superior temporal areas are unknown, research suggests that between ~60–150 ms, the brain encodes spoken word information at a sublexical level. The timing and location of the peak for spoken words in the present study is consistent with the majority of this previous work.

To date, there have not been any investigations into an analogous stage for sign encoding. In part, this may be due to the fact that most previous studies have used hemodynamic methods that do not afford sufficient temporal resolution to distinguish between early and late processing stages. During a time window analogous to the well-established encoding processes for written and spoken words, ASL signs showed an activity peak in right IPS, which was only marginally stronger than for English words. It is unclear whether such activity reflects linguistic encoding (analogous to sublexical amplitude envelope information in spoken language, for example) or quasi-gestural sensory characteristics related to space and motion (Decety and Grèzes, 1999; Grossman and Blake, 2002; Malaia et al., 2012). The early right IPS activity has multiple possible interpretations, and may not be related to the fact that the stimuli were signed words, but rather to the proficiency of the participants in ASL. While prior studies have not found right IPS to be modulated by language proficiency, the participants in those studies have typically possessed higher proficiency in L2 (Leonard et al., 2010, 2011). In case studies of deaf signers with scant proficiency in any language, we have observed right IPS activation later at 300–350 ms (Ferjan Ramirez et al., 2013a). It is possible that modality and proficiency interact in parietal regions, perhaps reflecting a neural processing strategy that is uniquely useful for the dynamic visual linguistic content of sign languages. To fully disentangle these effects, and to unambiguously identify the analogous word encoding stage for sign languages, it will be necessary to conduct studies with native deaf and hearing signers and low proficiency deaf and hearing signers using carefully controlled stimuli that separate linguistic and sensory levels of processing [similar to recent work with spoken words (Travis et al., in press)]. These experiments are yet to be carried out, and the present results provide both anatomical and functional brain regions that can be used to test the interaction between proficiency and modality.

The results for word meaning and higher-level language encoding processes were more definitive and demonstrated that proficiency effects translate across spoken, written, and signed words. Beginning at ~200 ms, all three word types were processed in a highly similar left-lateralized network including inferior frontal, superior temporal, and anteroventral temporal areas. These regions have been hypothesized to provide core support for lexico-semantic encoding at a supramodal level (Marinkovic et al., 2003; Lambon Ralph et al., 2010), and are the main neural generators of the N400 response (Halgren et al., 1994; Marinkovic et al., 2003), even in infants who are only beginning to acquire language (Travis et al., 2011). These areas all showed semantic modulation in the congruent/incongruent picture-word matching task (albeit to a lesser extent for ASL). Analyses of the overall magnitude of the response to words in each language and modality showed that spoken, written, and signed words all evoke strong activity in these regions, consistent with previous intracranial recordings showing locally-generated differential responses to different semantic categories of words in the anterior temporal lobe, regardless of modality (Chan et al., 2011). Previous hemodynamic studies have found activity related to lexico-semantic processing in these areas for sign language (Petitto et al., 2000; MacSweeney et al., 2008; Mayberry et al., 2011), and N400 responses have been observed to sign (Neville et al., 1997). To our knowledge, however, this is the first demonstration of such activation patterns after so little L2 instruction in ASL. These findings provide strong support for the hypothesis that these areas, especially in the anterior temporal lobe, function as supramodal hubs for high-level semantic representations (Patterson et al., 2007; Visser and Lambon Ralph, 2011), and seem difficult to explain as reflecting either knowledge of unique entities or social conceptual knowledge (Simmons and Martin, 2009).

The different patterns we observed for the congruent-incongruent subtraction and the grand average of all activity provide a window into the nature of lexico-semantic processing. Up to this point, we have focused on modality-specific effects. We now turn to how the design of this study provides insights into the role of language experience on the neural processing of words. The participants had an average of almost 23 years of extensive experience with spoken English, approximately 19 years of experience with written English, but only a few months of primarily classroom instruction in ASL. Proficiency has profound effects on neural activity, and even on brain structure (Maguire et al., 2000). Numerous studies have demonstrated experience-related differences in bilingual language processing (Abutalebi et al., 2001; Chee et al., 2001; Perani and Abutalebi, 2005; Leonard et al., 2010, 2011; van Heuven and Dijkstra, 2010). These studies further show that a surprisingly small amount of L2 exposure is required to elicit automatic lexico-semantic processing (McLaughlin et al., 2004). The present results demonstrate that this is true for beginning L2 learning of ASL as well.

As would be expected, an examination of the lexico-semantic effects in the present study indicates that proficiency-modulated activity also occurs in sign processing. In particular, we found that ASL words evoked greater grand average activity than both spoken and written English in a network of mostly right hemisphere regions (the two left hemisphere regions that were significant in the grand average, IFG and IT, were not significant when congruent and incongruent trials were analyzed separately). It is striking that some of these areas (right LOT, pSTS, and IFG) are nearly identical to those that showed a non-dominant > dominant pattern in hearing Spanish-English bilinguals (Leonard et al., 2010, 2011). The results for L2 ASL learners provide additional evidence that these areas play an important role in processing words in a less proficient language. The present results, together with our previous findings, demonstrate that word processing in a less proficient L2 shows increased activity in these regions (particularly for semantically incongruent words) relative to word processing in the native language. The recruitment of these areas for both spoken and sign language L2 processing indicates that they function as an additional supramodal resource for processing meaning in a non-dominant language.

The dissociation between semantic congruity and overall activity across languages provides a finer-grained characterization of how proficiency affects neural processing. The English > ASL congruity effects in left fronto-temporal areas could suggest shallower or less complete processing of semantic content in the non-dominant language. The slower reaction times and lower accuracy for ASL support this hypothesis. However, given that subjects performed the task relatively well indicates that some neural processing strategy was used successfully. The ASL > English responses in the grand average MEG activity across both hemispheres suggest that additional neural resources were recruited to perform the task, although perhaps not at the same semantic depth. The overall stronger ASL > English differences for incongruent words compared to congruent words support this hypothesis. As these L2 learners improve their ASL proficiency, we predict that the grand average activity will decrease to English-like levels, and the congruent/incongruent difference will likewise increase. This represents a testable hypothesis for tracking neural processing strategies during development (Schlaggar et al., 2002; Brown et al., 2005) and later language acquisition in a bilingual context.

### Conflict of interest statement

The authors declare that the research was conducted in the absence of any commercial or financial relationships that could be construed as a potential conflict of interest.
